# Prevalence of Chronic Kidney Disease and Its Related Risk Factors in Elderly of Southern Iran: A Population-Based Study

**DOI:** 10.5402/2013/427230

**Published:** 2013-07-07

**Authors:** Leila Malekmakan, Parviz Khajehdehi, Maryam Pakfetrat, Alireza Malekmakan, Hamideh Mahdaviazad, Jamshid Roozbeh

**Affiliations:** ^1^Shiraz Nephro-Urology Research Center, Shiraz University of Medical Sciences, Shiraz 7193636154, Iran; ^2^Internal Medicine, Shiraz Nephro-Urology Research Center, Shiraz University of Medical Sciences, Shiraz 7193636154, Iran; ^3^VUMC, Amsterdam, The Netherlands; ^4^Social Determinat of Health Research Center, Shiraz University of Medical Sciences, Shiraz, Iran

## Abstract

*Aim*. The prevalence of chronic kidney disease (CKD) as a serious public health problem is growing in the elderly. This study aimed to assess CKD prevalence and its related risk factors in elderly population of Fars province. *Methods*. In this cross sectional study a total of 1190 elderly people are enrolled, and demographic and medical data were obtained. Data were analyzed by SPSS, and *P* of less than 0.05 was considered as statistically significant. *Results*. Prevalence of CKD stages III–V was 27.5% in the 60–69 years age group, 36.5% in the 70–79 years age group, and 40% in the ≥80 years age group. The prevalence of CKD increased with ageing in both men and women. Female gender was the strongest risk factor for CKD. *Conclusions*. Prevalence of CKD in elderly is high in Southern Iran, which has become an important health problem while it can be prevented or delayed in progression.

## 1. Introduction

The prevalence of chronic kidney disease (CKD) as a serious public health problem is growing in the elderly [1, 2]. CKD is associated with end-stage renal disease (ESRD) and increases morbidity and mortality and cost of the health care system [3–5].

CKD is defined as either kidney damage, estimated by using such markers as albuminuria, or estimated glomerular filtration rate (eGFR) less than 60 mL/min/1.73 m^2^ [3].

In persons 60 years and older, approximately 30% have albuminuria and 26% have GFR of less than 60 mL/min/1.73 m^2^. Also elderly population is susceptible to kidney damage from other chronic diseases such as hypertension, diabetes mellitus, and tubulointerstitial disorders [6]. So early diagnosis and implementation of therapeutic strategies have been emphasized to delay the progression of disease and increase quality of life in these patients [7].

Although high prevalence of CKD in elderly, few studies have especially addressed this problem. Also there is no specific study about CKD prevalence in elderly and its related risk factors in our area. This population-based study aimed to assess the CKD prevalence and its related risk factors in elderly population of Fars province, Southern Iran.

## 2. Methods and Materials

### 2.1. Study Population

This is a cross sectional, population-based study in Southern Iran. A total of 1190 elderly persons (age ≥ 60) were enrolled during a 2-year period (September 2009 to December 2011). This study complies with the Declaration of Helsinki and was approved by the local Ethics Committee. All patients gave written informed consent.

### 2.2. Sample Size

For calculation of sample size, with estimation error of 2.5% and a 95% confidence interval, we obtained a sample size of 1153. According to a multistage stratified cluster random sampling, Southern Iran was subdivided to geographical areas. After selecting randomly a city from each area, that is divided into urban and rural parts, used by the population and covered by its health center, and based on population proportional, the required sample size was chosen. Finally, we collected 1190 persons that were invited to visit the local health center, and detailed evaluation was done.

### 2.3. Measurements

All subjects completed a data collection form by face-to-face interview including demographic data and medical history. Physical examinations (measurement of height, weight, and blood pressure) and specific laboratory tests were undertaken by trained health providers. Blood samples for measuring the level of serum creatinine (SCr) were collected from the antecubital vein, and a spot urinary protein was assessed for proteinuria using dipsticks. Blood specimens were centrifuged on site and transported with spot urine samples to the reference laboratory in Shiraz. All urine and blood samples were analyzed with the same equipment throughout the duration of the study.

### 2.4. Definitions

#### 2.4.1. CKD

The severity of CKD can be classified into 5 stages by the Kidney Disease Outcome Quality Initiative (K/DOQI) guideline [11, 12]: stage 0: eGFR > 90 mL/min/1.73 m^2^, and; no proteinuria; normal kidney function, stage 1: eGFR > 90 mL/min/1.73 m^2^ and with evidence of kidney damage, stage 2: eGFR 60–89 mL/min/1.73 m^2^; mild decrease in GFR; stage 3: eGFR 30–59 mL/min/1.73 m^2^ and moderate decrease in GFR; stage 4: eGFR 15–29 mL/min/1.73 m^2^ and severe decrease in GFR; and stage 5: eGFR < 15 mL/min/1.73 m^2^ or in dialysis, kidney failure. 

According to this guideline, in this study CKD is defined as either kidney damage or GFR < 60 mL/min/1.73 m^2^.

#### 2.4.2. eGFR

For this study, eGFR was estimated using Modification of Diet in Renal Disease (MDRD) equation [13, 14]: eGFR = 186 × (SCr)^−1.154^ × (Age)^−0.203^ × (0.742  if  female).

In this equation, GFR and SCr are expressed as mL/min per 1.73 m^2^ and mg/dL, respectively. 

#### 2.4.3. Proteinuria

Proteinuria was estimated using visually read dipsticks. None and trace urinary protein were classified as no proteinuria and the rest of them (1+, 2+, and 3+) as proteinuria. 

#### 2.4.4. Body Mass Index (BMI)

 Weight (in kilograms) and height (in meters) were used to calculate BMI, which was categorized as 3 groups of <18.5 kg/m2, 18.5 to 24.9 kg/m2, and ≥25 kg/m^2^.

#### 2.4.5. Hypertension (HTN)

It was defined as systolic blood pressure (SBP) > 130 mm Hg or diastolic blood pressures (DBP) > 80 mm Hg, also patients who had a positive history of HTN and were receiving antihypertensive drug(s). Blood pressure was measured 2 times after resting for at least 15 min using standard adult mercury sphygmomanometer. The average of the two readings was finally recorded.

### 2.5. Statistical Analysis

Data were analyzed by the Statistical Package for the Social Sciences software version 15.0 (SPSS Inc. Chicago, IL, USA). Qualitative data are expressed as number and percentage, that are analyzed by the chi-square test. Quantitative data were presented as mean and standard deviation. The ordinal regression test was used to determine the risk factors associated with different CKD stages. A *P*-value of less than 0.05 was considered as statistically significant. 

## 3. Results

In this large population-based study, we enrolled 1190 subjects aged ≥60 years that were randomly selected from the general population of Southern Iran, Fars province. 

Table 1 shows the main categories of CKD and baseline characteristics of them. Mean age of all subjects was 67.5 ± 6.8 years. The subjects in CKD stages 0–II were younger than subjects in CKD stages III–V (66.9 ± 6.1 versus 68.7 ± 7.0, *P* < 0.001). Of the total study population, 40.4% were males and 59.6% were females; in CKD stages III–V 78.2% were females and 21.8% were males (*P* < 0.001). In categories of occupation and education most subjects were housekeepers (39.2%) and under diploma (70.9%). BMI was categorized in three groups: <18.5 kg/m^2^, 18.5 to 24.9, and ≥25 kg/m^2^. Nearly half of subjects in CKD stages III–V were overweight. HTN was detected in 38% of subjects, which in category of CKD stages III–V this percentage was 49.6%. 

Table 2 demonstrates the comparison of different stages of CKD between genders; the percentages of CKD stage 0-I were higher in men, while the percentages of CKD stages II, III, and IV were higher in women. 

Table 3 shows prevalence of CKD stages III–V in different age groups within genders. Overall prevalence of CKD stages III–V based on eGFR < 60 mL/min/1.73 m^2^, calculated with MDRD equation, was 31.2%. Prevalence of CKD stages III–V were 27.5% in the 60–69 years age group, 36.5% in the 70–79 years age group, and 40% in the ≥80 years age group. The prevalence of CKD increased with ageing in both males and females (*P* = 0.002). The prevalence of CKD stages III–V was highest among women within the age group of ≥ 80 years (61.9%).

According to Table 4, female gender was the strongest risk factor for CKD (adjusted OR: 3.22, 95% CI: 1.49–3.7). The second most important risk factor for CKD was HTN (adjusted OR: 1.75, 95% CI: 1.31–2.33). Finally, age (adjusted OR: 1.05, 95% CI: 1.03–1.07) and BMI (adjusted OR: 1.05, 95% CI: 1.01–1.09) were other significant risk factors in subjects with CKD. 

## 4. Discussion

Elderly people are an important part of the population, which increased dramatically. There is a shift to older-in-age distribution that has begun in developing countries including in Iran [15]. Despite this fact that receiving to advanced age is considerable, morbidity and disability rates that be occurred, could be challenging issue [16]. In this respect, CKD is predominantly a disease of older people, because cumulative exposure to causes of CKD increases with age [17]. Progression of CKD to renal failure requires expensive renal replacement therapies [18], hemodialysis being the most common of them in our center [19]. Although the current concept suggests that if CKD is detected and treated early its adverse outcomes could be delayed or even prevented, unfortunately information about CKD in the elderly population has been poorly studied in developing countries especially in Iran.

### 4.1. Prevalence of CKD

The report of CKD prevalence in elderly varies significantly from country to country, from 15.8% in China [5] to 35.8% in Finland [1]. In this study, the CKD prevalence in our elderly population was 31.2%, that is roughly in line with data from other studies with the same definition and method [1, 2, 4, 10]. Also in this study like other same surveys [2, 5, 20], the increase in CKD prevalence with increasing age was observed in both sexes; therefore, the subjects with CKD stages III–V were significantly older than those with stages 0-II. The growing prevalence of decreased renal function in older persons can be due to an increase in age-related risk factors for progression to the CKD [20]. Aging undergoes several changes in body that impact kidney function, so GFR declines with age [7]. 

### 4.2. Characteristics-Different and Risk Factor in CKD, (Table 5)

A gender-different prevalence of CKD was discovered in most related studies. In concordance to other studies [2, 4, 5, 10, 20], our study showed a higher prevalence of CKD in women compared with men. So the female gender was the strongest risk factor for CKD in the current study. Overall we have seen that in each age group the prevalence of CKD in women was higher than men. It may be a result of the difference between women and men in glomerular structure, glomerular haemodynamics, muscle mass, and the hormone metabolism [21, 22]. Additionally, these days the higher CKD prevalence in women might be caused by lower physical activity and high prevalence of cardiometabolic risk factor.

We found the prevalence of CKD stages III–V to be significantly higher in housekeeping wives and in those under diploma. A similar study in old age showed that CKD is more prevalent in those who had lower education level, but it is not significant [2]. It may have resulted from the possibility of low level of information about prevention of risk factor of renal problem in housekeeping wives as whom that had low education. 

We found that the most important risk factors for CKD were HTN and BMI; was this significant association seen in the previous studies [3, 20, 23]. Previous report of our center disclosed that diabetes mellitus is the most common cause of ESRD [24]; likewise, we found that Preparing interventions including improvement of knowledge level, developed appropriate guidelines for modifying life style, and strategies for obesity, HTN and diabetes mellitus prevention or control as a common risk factor of CKD can be useful.

In our study, the following limitations should be considered. First, CKD prevalence strongly depended on the used equation for GFR estimation especially in elderly persons; some studies report that the MDRD equation in comparison with the Cockcroft-Gault formula adjusted by body surface area systematically underestimates GFR in healthy populations, so careful modification of MDRD equations may be necessary in Iranian populations with CKD. Second, we were not able to take more underlying risk factors of CKD, such as cardiovascular disease, diabetes, infection, and socioeconomic factors, into account in this study. Third, due to nature of study design, we could not report the causal relationship between CKD and related risk factors. Finally, Fars province is one of the thirty-two provinces in Iran, so the prevalence of CKD reported in this study could not be generalized to the whole Iranian elderly populations. 

We conclude that prevalence of CKD is high in Southern Iran, and it is already a common disease in the worldwide general population. Epidemiological data about CKD provide a helpful approach for prevention and treatment in the studied population. 

Our findings indicate the importance of a lifestyle plan and intervention programs for prevention and delayed course of CKD and its complications in Southern Iran. In addition, it is set to support strategies for early detection of CKD and its risk factors.

## Figures and Tables

**Table 1:** Demographic characteritics of participants with chronic kidney diseases according to their stages.

Variables		CKD stages^†^		
	0 to II (%)	III to V (%)	*P*-value	Total (%)
	(*n* = 819)	(*n* = 371)		(*n* = 1190)
Age (mean ± SD)	66.9 ± 6.1	68.7 ± 7.0	<0.001	67.5 ± 6.8
Age groups (years)				
60 to 69	537 (65.6)	204 (55.0)	0.002	741 (62.3)
70 to 79	231 (28.2)	133 (35.8)		364 (30.6)
≥80	51 (6.2)	34 (9.2)		85 (7.1)
Sex				
Male	400 (48.8)	81 (21.8)	<0.001	481 (40.4)
Female	419 (51.2)	290 (78.2)		709 (59.6)
Occupation				
Governmental	17 (2.1)	6 (1.6)		23 (1.9)
Housekeeper	263 (32.1)	204 (55.0)	<0.001	467 (39.2)
Farmer	109 (13.3)	20 (5.4)		129 (10.8)
Self-employed	149 (18.2)	38 (10.2)		187 (15.7)
Industrial	1 (0.1)	1 (0.3)		2 (0.2)
Others	37 (4.5)	37 (10.0)		155 (13.1)
Missing	243 (29.7)	65 (17.5)		227 (19.1)
Education				
Under diploma	564 (68.9)	279 (75.2)	0.05	843 (70.9)
Diploma and associated degree	62 (7.6)	23 (6.2)		85 (7.1)
Bachelor and higher	14 (1.7)	1 (0.3)		15 (1.3)
Missing	179 (21.8)	68 (18.3)		247 (20.7)
BMI^‡^				
Under 18.5	23 (2.8)	6 (1.6)	<0.001	29 (2.4)
18.6 to 25	316 (38.6)	112 (30.2)		428 (36.0)
Up to 26	298 (36.4)	184 (49.6)		482 (40.5)
Missing	182 (22.2)	69 (18.6)		251 (21.1)
Hypertension*				
No	551 (67.3)	187 (50.4)	<0.001	738 (62.0)
Yes	268 (32.7)	184 (49.6)		452 (38.0)

^†^CKD classified into 5 stages; stage 0: eGFR >90 mL/min/1.73 m^2^, no proteinuria, and normal kidney function; stage 1: eGFR >90 mL/min/1.73 m^2^ with evidence of kidney damage; stage 2: eGFR 60–89 mL/min/1.73 m^2^, mild decrease in GFR; stage 3: eGFR 30–59 mL/min/1.73 m^2^, moderate decrease in GFR; stage 4: eGFR 15–29 mL/min/1.73 m^2^, severe decrease in GFR; and stage 5: eGFR <15 mL/min/1.73 m^2^ or in dialysis, kidney failure.

^‡^Body mass index was defined as weight (in kilograms) and height (in meters), which was categorized as 3 groups of <18.5 kg/m^2^, 18.5 to 24.9 kg/m^2^, and ≥25 kg/m^2^.

*Hypertension was defined as SBP >130 mmHg, DBP >80** **mmHg, or those who had a positive history of hypertension and were receiving antihypertensive drugs.

**Table 2:** Frequency of chronic kidney disease (CKD) stages in male and female.

Variables	Male (%)	Female (%)	Total (%)	*P*-value
	(*n* = 481)	(*n* = 709)	(*n* = 1190)	
Stage 0	40 (8.3)	13 (1.8)	53 (4.5)	<0.001
Stage I	19 (4.0)	3 (0.4)	22 (1.8)	<0.001
Stage II	341 (70.9)	403 (56.8)	744 (62.5)	<0.001
Stage III	79 (16.4)	286 (40.3)	365 (30.7)	<0.001
Stage IV	4 (0.3)	3 (0.4)	3 (0.4)	0.651
Stage V	2 (0.2)	1 (0.1)	1 (0.1)	1.000

**Table 3:** Comparison prevalence of chronic kidney disease (CKD) stages III–V in age groups in relation to genders.

Age groups*	Males**		Females**		Total	
	CKD number	Prevalence (%)	CKD number	Prevalence (%)	CKD number	Prevalence (%)
60–69	45	15.8%	159	34.8%	204	27.5%
70–79	28	18.2%	105	50.0%	133	36.5%
≥80	8	18.6%	26	61.9%	34	40.0%

Total	81	16.8%	290	40.9%	371	31.2%

*There is significant *P* value prevalence of CKD among different age groups (*P* = 0.002).

**There is significant *P* value prevalence of CKD between sex groups (*P* ≤ 0.001).

**Table 4:** Risk factors associated with different CKD stages based on ordinal regression test.

Variables	*β*	Adjusted odds ratio	95% CI^†^	*P* value
Age	<0.001	1.05	1.03–1.07	0.05
BMI	0.008	1.05	1.01–1.09	0.047
Sex				
Men	Baseline	1	—	—
Women	1.17	3.22	1.49	<0.001
Occupation				
Governmental	Baseline	1	—	—
Housekeeper	−0.288	0.75	0.26	0.591
Farmer	−0.971	0.38	0.13	0.075
Others	−0.811	0.44	0.16	0.11
Education				
Up to diploma	Baseline	1	—	—
Under diploma	0.261	1.3	0.79	0.307
Hypertension				
No	Baseline	1	—	—
Yes	0.558	1.75	1.31	<0.001

^†^CI: confidence interval.

**Table 5:** Prevalence of chronic kidney disease (CKD) in different studies.

Author [ref]	CKD definition, GFR^†^ formula	Age of study group	CKD prevalence
Malekmakan et al.—current study	Stage III–V, MDRD^‡^	>60 years	31.2%
Viktorsdottir et al. [8]	Stage III–V, MDRD	>60 years	Men: 11.4% Women: 27.1%
Rothenbacher et al. [2]	Stage III–V, MDRD	>65 years	34.3%
Li et al. [5]	GFR < 60 mL/min/1.73 m^2^ or presence of kidney damage (albuminuria ≥ 3), MDRD	>60 years	15.8%
Wasén et al. [1]	GFR < 60 mL/min/1.73 m^2^, MDRD	>64 years	35.8%
Hemmelgarn et al. [4]	GFR < 60 mL/min/1.73 m^2^	>66 years	35.4%
O'Riordan [9]	GFR < 60 mL/min/1.73 m^2^	>70 years	25%
Swedko et al. [10]	GFR < 50 mL/min/1.73 m^2^	>65 years	28.9%

^†^GFR: glomerular filtration rate. ^‡^MDRD: Modification of Diet in Renal Disease.
